# Effects of Hydrothermal Pretreatment on the Structural Characteristics of Organosolv Lignin from *Triarrhena lutarioriparia*

**DOI:** 10.3390/polym10101157

**Published:** 2018-10-16

**Authors:** Tianying Chen, Zhiwen Li, Xueming Zhang, Douyong Min, Yuying Wu, Jialong Wen, Tongqi Yuan

**Affiliations:** 1 Beijing Key Laboratory of Lignocellulosic Chemistry, Beijing Forestry University, Beijing 100083, China; tianyingchen15@163.com (T.C.); lizhiwen1018@163.com (Z.L.); xm_zhang@bjfu.edu.cn (X.Z.); wuyuying-1980@163.com (Y.W.); ytq581234@163.com (T.Y.); 2 Guangxi Key Laboratory of Clean Pulp and Papermaking and Pollution Control, Nanning 530004, China; mindouyong@gxu.edu.cn

**Keywords:** *Triarrhena lutarioriparia*, quantitative heteronuclear-single-quantum-coherence spectra (HSQC), hydrothermal pretreatment, organosolv, lignin

## Abstract

The effects of hydrothermal pretreatment (170–180 °C, 30–60 min) on the structural characteristics of enzymatic and extracted lignin from *Triarrhena lutarioriparia* (*TL*) during the integrated delignification process have been comprehensively investigated. Ion chromatography and NMR characterization showed that liquid products after mild hydrothermal process (170 °C, 30 min) were mainly composed of xylooligosaccharide (XOS) with different degrees of polymerization (DP ≥ 2). In addition, the structural changes of lignin during hydrothermal pretreatment and organic acid delignification process have been demonstrated by quantitative 2D heteronuclear single quantum coherence (2D-HSQC) and ^31^P-NMR techniques. Results showed that the structural changes of lignin (e.g., cleavage of β-*O*-4 linkages) induced by the hydrothermal pretreatment will facilitate the subsequent organic acid delignification process, and acetylated lignin could be obtained with a considerable yield, which can be used in lignin-based composite and candidate feedstock for catalytic upgrading of lignin. In short, the proposed process facilitates the producing of XOS and acetylated lignin for lignin valorization.

## 1. Introduction

With the depletion of fossil fuels, the current dependence on these non-renewable resources needs to be reduced. The substitution of fossil fuels by renewable energy is one of the most efficient ways to resolve this problem [1]. Lignocellulosic biomass as a sustainable, abundant, and environmentally friendly energy source, is a primary candidate for the substitution of fuels and chemicals [2]. However, the current utilization of lignocelluloses has mainly focused on the paper industry. Due to its changed and destructed structure, lignin from the pulping process is principally burned to generate the power [3]. In the current biorefinery process, there are various barriers, defined as biomass recalcitrance, for the effective conversion of biomass, such as the degree of lignification, the structural heterogeneity and complexity of cell-wall constituents as well as its complex cross-linking [4]. Therefore, it is necessary to develop a novel method to overcome the cell wall recalcitrance and obtain high-quality lignin for further lignin valorization.

Different kinds of pretreatments have been investigated and elaborately reviewed, including physical, chemical, and biological methods, and a combination of these methods [5,6]. It has been reported that pretreatment had pervasive impacts on the overall conversion scheme from choice of feedstock through to residue processing, and co-product potential [6]. Among these pretreatments, organic solvent-based pretreatment has been reported as a promising process for the effective separation of lignin due to its relatively mild conditions, high-efficiency, high-quality lignin fractionation, its ability to readily recover and be recycled, and its low input and high output in the factory [7]. Various organic solvents have been applied to the fractionation of different lignocellulosic biomasses in recent years [8]. If a solvent comes from biomass, it will be the most suitable for the separation and conversion of the biomass as an eco-friendly strategy. It has been reported that formic acid/acetic acid can be produced by hydrothermal oxidation of alkali lignin [9] or hydrolysis of xylans [10]. This has the benefit of being an easy recycling solution. Similarly, Abdelkafi et al. reported that the organic fractionation process was undertaken on Alfa grass by acetic acid/formic acid/water to produce lignin and to analyze the structure of lignin [11]. Meanwhile, formic acid was used as an effective agent to produce pulp and lignin by-product from bamboo, and acceptable delignification degrees (over 79.1%) have been achieved at 101 °C for 2–3 h via conventional heating [12]. However, hemicelluloses have not been effectively collected during the lignin isolation process, which probably impairs the overall economic efficiency of the biorefinery.

Hydrothermal pretreatment has been considered as a leading and promising pretreatment technology [13]. It has been demonstrated that pretreatment can hydrolyze and dissolve a great part of the hemicelluloses due to the emergence of acetic acid from xylans. Recently, the continual flow of hot water through a lignocellulosic biomass showed that it is possible to enhance the entire performance of hot water pretreatment [14]. In general, the hemicelluloses in the feedstock can be hydrolyzed into xylooligosaccharides (XOS, degree of polymerization, DP 2–6) and xylose during hydrothermal pretreatment, which was dependent on the pretreatment severity and the purpose of the process. Wen et al. reported that bamboo culms treated with a hydrothermal process at 180 °C for 30 min could produce XOS (degree of polymerization, DP 2–6) [15]. Meanwhile, hydrothermal pretreatment also has similar efficiency on other grasses, such as wheat straw [16] and sweet sorghum [17]. Furthermore, it has been reported that hydrothermal pretreatment can facilitate the different delignification processes, such as kraft pulping [10], organic acid delignification [15], and alkaline delignification [18]. However, the effects of different hydrothermal pretreatments on the structural characteristics of lignin in the substrates and subsequent delignification process should be further investigated. 

*Triarrhena lutarioriparia* (*TL*) is an abundant, low-production cost energy crop. Thus, it draws considerable attention and is viewed as an interesting raw material for the industrial bioconversion processes [19]. In the present study, hydrothermal pretreatment was performed on *TL* under various conditions (170 °C for 30 min, 170 °C for 60 min, and 180 °C for 30 min) to investigate the effects of hydrothermal pretreatments on the yield and structural characteristics of the isolated products (mainly XOS) and hydrothermally-treated residual substrates. Subsequently, the effects of hydrothermal pretreatment on the structural features of lignin obtained by organic acid treatment were evaluated by composition analysis, delignification ratio, 2D heteronuclear single quantum coherence (2D-HSQC), and ^31^P-NMR spectroscopy. It is believed that the results will enhance the understanding of lignin structure in *TL* and extend the availability of the combined process in the current biorefinery industry.

## 2. Materials and Methods

### 2.1. Materials

*Triarrhena lutarioriparia* (*TL*) was collected from National Experiment Station for Precision Agriculture, Xiaotangshan, Beijing, China. The stems were air dried and further ground to obtain the fractions with sizes between 40 and 60 mesh. The fractions were extracted with toluene-ethanol (2:1, *v*/*v*) for 8 h to remove the wax, and with hot water (80 °C) for 2 h to remove the starch. Subsequently, the extract-free samples were oven-dried and stored in a sealed bag before use. All chemicals used in this study were analytical grade and purchased from Sigma Chemical Co. (Beijing, China). The cellulase (Cellic^®^ CTec2, 100 FPU/mL) was bountifully provided by Novozymes (Beijing, China). 

### 2.2. Fractionation Process

*TL* was performed on different hydrothermal conditions to isolate hemicelluloses and then extracted with organic acid to obtain lignin fractions. Typically, 7 g extract-free *TL* was submitted to a 100 mL stainless steel batch reactor (Sen Long Instruments Company, Beijing, China) with 70 mL deionized water. The reaction was carried out at 170 °C for 30 min. After completion, the mixture was cooled and filtered to obtain a solid residue and filtrate. The solid residue was washed with water to be pH neutral and then further dried at 60 °C until it was a constant weight, which was then labeled as P170-30. The filtrate of about 2 mL was directly taken out for the detection of the liquid products (XOS and inhibitor) to estimate the effect of hydrothermal pretreatment. Meanwhile, the filtrate and washing solution were mixed, concentrated, and freeze-dried to obtain the liquid products (hemicelluloses or XOS, labeled as H170-30). The hydrothermally-pretreated residue was further treated by organic acid (formic acid/acetic acid/water, 3/5/2, *v*/*v*/*v*) with a solid to liquid ratio (1:10) in a flask with three necks at 60 °C oil-bath for 1 h, followed by 107 °C for 3 h. After the reaction, the mixture was filtered and washed until it was pH neutral to obtain lignin-containing filtrate and cellulose-rich residue. The filtrate and washing liquid were combined and concentrated by a rotary evaporator to about 40 mL, and then the concentrated solution was poured into 10 volumes of acidic water (pH = 2, adjusted with HCl) to precipitate organic organosolv lignin fractions. The precipitation was collected and washed with acidic water by centrifugation followed by drying to obtain L170-30. The residue was dried and the final product labeled as OP170-30. After changing the detailed condition of hydrothermal pretreatment, different products were obtained. Detailed conditions of the experiment are shown in the Supplementary Material, Table S1. The experiments were run in duplicate and the data reported are average values.

### 2.3. Preparation of DEL Samples from Raw and the Hydrothermally-Treated Substrates

Double enzymatic lignin (DEL), which can represent the overall structural feature of lignin in the plant cell wall and the pretreated substrates [20], was used to investigate the influence of hydrothermal pretreatment on lignin fractionation and structural characteristics. The detailed procedures for preparation of DEL from *TL* and the pretreated *TL* substrates were similar to that reported in our previous publication [20]. Finally, the enzymatic lignin in the *TL* and the hydrothermally-treated *TL* substrates were labeled DEL, PDEL170-30, PDEL170-60, and PDEL180-30, respectively.

### 2.4. Characterizations of the Isolated Products

The analysis of the chemical compositions of *TL* and the pretreated *TL* substrates after different hydrothermal and organic acid treatments were conducted according to the National Renewable Energy Laboratory (NREL) standard analytical procedure [21]. In addition, the contents of carbohydrates in the lignin preparations were determined as previously reported in Reference [20]. 

To clarify the effect of different hydrothermal conditions on the degraded products, the quantification of xylose, XOS (DP, 2–6 and DP > 6), inhibitory products (formic acid, acetic acid, lactic acid, furfural, and 5-hydroxymethylfurfural (HMF)), and hemicelluloses was necessary. All the monosaccharide and XOS were measured by the Ion Chromatography (HPAEC Dionex, ICS 3000, Sunnyvale, CA, USA) as previously reported in Reference [22]. Specifically, oligosaccharides (DP > 6) are essential for evaluating the degradation of hemicelluloses, and its content can be determined based on previous literature [23,24]. The inhibitory products could inhibit the further fermentation process of cellulose. These products were quantified by HPLC (Agilent 1200 series, Agilent Technologies, Palo alto, CA, USA), equipped with an Aminex HPX-87H analytical column (Bio-Rad, Hercules, CA, USA) and with a refractive index detector and an ultraviolet detector at 254 nm. Furthermore, the structural characteristics of low DP hemicelluloses or XOS in the filtrate after hydrothermal pretreatment were ascertained with 2D-HSQC spectra as previous paper [25]. 

The molecular weights of the lignin samples were performed by gel permeation chromatography (GPC, Agilent 1200, USA) with an Agilent UV detector (240 nm) on a PL-gel 10 mm Mixed-B 7.5 mm ID column and calibrated with narrow molecular weight distribution polystyrene standards (1390, 4830, 9970, 29,150). Tetrahydrofuran (THF) of HPLC grade was used as the mobile phase. Traditionally, about 2 mg of the lignin was dissolved in 1 mL THF. Then, 20 µL of the solution was injected into the system. Due to the limit of solubility of native lignin in THF, an acetylation process was necessary for DELs samples. The detailed procedure for acetylation is provided in the Supplementary Material. The NMR spectra of the lignin samples were recorded to define the structural changes of lignin during different treatments. For quantitative 2D-HSQC spectra, 40 mg lignin was dissolved in 0.5 mL DMSO-*d*_6_ on a Bruker AVIII 400 MHz spectrometer at 25 °C, and the pulse sequence “hsqcetgpsi” was selected from Bruker Standard Pulse Library. The quantitative ^31^P-NMR spectra of the lignin samples were detected by the reaction of lignin with 2-chloro-4,4,5,5-tetramethyl-1,3,2-dioxaphospholane (TMDP) according to previous literature [26,27].

## 3. Results and Discussion

### 3.1. Chemical Composition of the Substrates and Lignin

**Figure 1:**
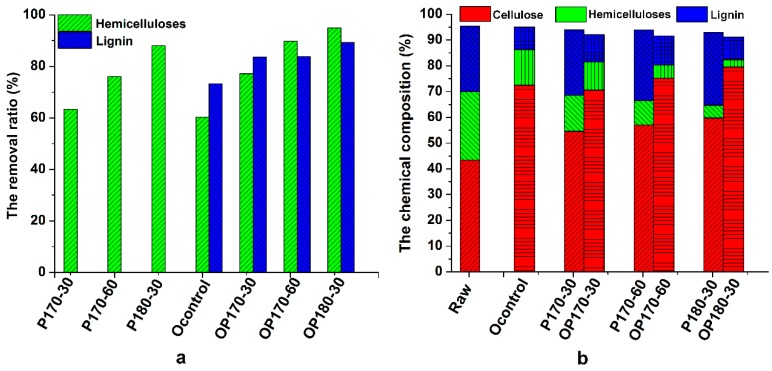
(**a**) The removal ratio of hemicelluloses and lignin; (**b**) chemical compositions of untreated *Triarrhena lutarioriparia* (*TL*) and the treated residues.

It is indispensable to evaluate the effects of various treatments on the chemical composition of the substrates after treatment. Thus, the removal ratio of hemicelluloses and lignin, as well as the chemical compositions of the untreated and treated *TL*, are plotted in Figure 1. As shown in Figure 1a, the removal ratio of hemicelluloses during hydrothermal pretreatment displayed an increasing trend with the aggravation of treatment condition from 170 °C for 30 min to 170 °C for 60 min, and further to 180 °C for 30 min. Meanwhile, subsequent organic acid treatment led to an elevation of the corresponding removal ratio of hemicelluloses, reaching 94.93% at 180 °C for 30 min. The removal ratio of hemicelluloses (77.14–94.93%) in OP170-30 to OP180-30 was apparently higher than that (60.26%) of Ocontrol, implying that hydrothermal pretreatment facilitates the removal of hemicelluloses under the conditions given. Meanwhile, the delignification ratio of the control experiment (Ocontrol) reached 73.25%, which was slightly lower than those of hydrothermally-treated residues (OP170-30, OP170-60, and OP180-30). In addition, the delignification ratio (89.34%) of OP180-30 was higher than that (83.72%) of OP170-30, implying that the elevation of pretreatment temperature provides a boost for deligninfication process.

The chemical compositions of the raw material and the treated residues are shown in Figure 1b. The content of cellulose, hemicelluloses, and lignin in *TL* was 43.19%, 26.63%, and 25.43%, respectively. After the hydrothermal pretreatment, the proportion of hemicelluloses was continually decreased with the increased pretreatment severity. Similarly, the hemicelluloses proportion in the substrate after the hydrothermal and delignification process was further reduced as compared with that in P170-30 to P180-30. In general, the removal ratio of lignin and hemicelluloses is the important index to evaluate the effectiveness of the pretreatment. However, the purity, molecular structure, and chemical reactivity also need to be considered before lignin valorization.

With respect to lignin purity, the associated carbohydrates in the obtained lignin were analyzed to evaluate the purity of lignin obtained after these processes. As shown in Table 1, the major sugars in PDELs were glucose followed by xylose, together with a small percentage of arabinose, galactose, and mannose, while DEL presents the highest xylose content, suggesting that the majority of hemicelluloses were removed after hydrothermal pretreatment, which is in accordance with the result from GPC analysis. Meanwhile, the content of carbohydrates in Lcontrol was significantly lower than that of DEL, especially xylose and glucose, suggesting that a small amount of sugar was extracted with the lignin by organic acid. By contrast, the content of carbohydrates in L180-30 to L170-30 is low (from 2.19% to 2.95%) because the possible lignin–carbohydrates linkages were further broken under the organic acid treatment.

### 3.2. Characterizations of Liquid Products after Hydrothermal Pretreatment

**Figure 2:**
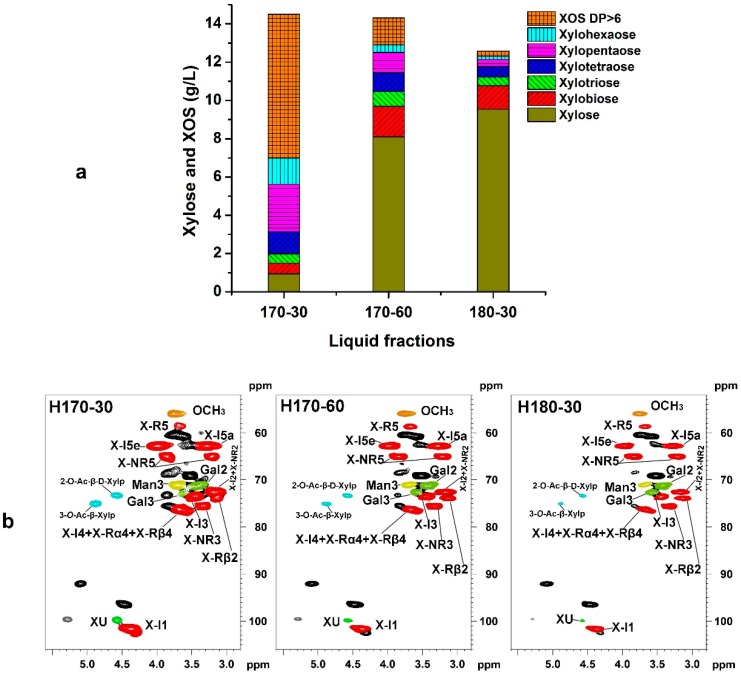
The analysis of liquid products: (**a**) the concentrations of xylose and xylo-oligosaccharides; (**b**) 2D heteronuclear single quantum coherence (2D-HSQC) spectra of the liquid products (hemicelluloses or xylooligosaccharide (XOS)).

During the hydrothermal pretreatment, abundant degraded products were released in the liquid fraction. To evaluate the effect of hydrothermal pretreatment on the basic characteristics of the filtrate fraction, it is essential to comprehensively investigate the composition and content of liquid products. It has been reported that the severity of hydrothermal treatment (log R_0_, 3.51–4.44) corresponds to the reaction condition from 160 °C for 30 min to 190 °C for 30 min [28]. This demonstrated that the oligosaccharides were mainly released among these ranges in this study. The DP distribution and the concentration of XOS under different pretreatment conditions are shown in Figure 2a. Obviously, the distribution of xylose and XOS with different degrees of polymerization (DP) was affected by the pretreatment time and temperature. In detail, the concentration of high-DP (DP > 6) XOS was remarkably decreased from 7.50 g/L in 170-30 to 0.28 g/L in 180-30, while the concentration of xylose was significantly increased from 0.95 g/L in 170-30 to 9.54 g/L in 180-30 with the prolongation of reaction time (170 °C for 60 min) and the elevation of pretreatment temperature (180 °C for 30 min), which is mainly attributed to the rapid hydrolysis of xylan or XOS at high severity. Meanwhile, the concentrations of xylobiose (from 0.55 to 1.58 g/L) and xylotriose (from 0.49 to 0.78 g/L) also displayed an increasing trend, while that of XOS (DP ≥ 4) were decreased with the extension of reaction time (from 170-30 to 170-60). However, the concentrations of XOS (DP ≥ 3) in 180-30 were decreased as compared with that of 170-30. The total concentrations of xylose and XOS (DP ≥ 2) also declined from 14.50 g/L to 12.58 g/L with the aggravation of pretreatment severity, which can likely be ascribed to the formation of byproducts from monosaccharides under harsh severity.

It has been reported that the hydrolyzed monosaccharides from hemicelluloses can be transformed into some byproducts, such as furfural, HMF, and organic acid [6] which inhibit yeast fermentation in bioethanol production [29]. In the present work, the concentrations of the main degradation products are listed in Table 2. Among them, acetic acid was obtained from the cleavage of the acetyl groups in acetylated hemicelluloses [30]. The amount of acetic acid rapidly increased from 1.67 to 3.86 g/mL with the increase of pretreatment severity, and its content was still the highest among all the degraded products, which is in accordance with previous Reference [28]. The concentration of furfural ranged from 0.12 to 4.09 g/L with the enhancement of pretreatment severity. The highest concentration of furfural was formed at the strongest severity (180 °C for 30 min). By contrast, the HMF was slightly presented in all the liquid products obtained from different hydrothermal conditions, especially for the condition at 170 °C for 30 min. Although the formation of HMF increases with retention time and temperature, the concentration of HMF was low (0.21 and 0.41 g/L). Meanwhile, the degradation of furfural and 5-hydroxymethylfurfural under acidic condition can promote the generation of formic acid during the hydrothermal treatment [31]. Results also showed that the formation of formic acid was aggravated with the increased pretreatment severity. In short, considering the relatively low yield of xylose and XOS (DP > 2) as well as the high content of byproducts under high pretreatment severity, it could be concluded that the pretreatment should be performed under mild conditions. The 2D-HSQC spectra were also conducted to analyze the structural characteristics of liquid products, and the corresponding spectra are shown in Figure 2b. The signals for XOS have been assigned and marked based on previous literature [25,32]. The methoxyl group (OCH_3_) was easily identified at δ_C_/δ_H_ 56.1/3.75. The (1→4)-*β*-d-Xylp marked as X (shown in red) are the predominant contours in these spectra. In detail, the internal xylan correlation peak (X-I1, X-I2, X-I3, X-I4, X-I5a, and X-I5e) from the backbone was located at δ_C_/δ_H_ 101.6/4.36 (C_1_/H_1_), 72.6/3.18 (C_2_/H_2_), 73.4/3.45 (C_3_/H_3_), 76.2/3.67 (C_4_/H_4_), and 62.8/3.99 and 3.28 (C_5_/H_5_). Coincidently, non-reducing-end xylan residues (X-NR2) also shared the same chemical shifts with X-I2. Other non-reducing-end peaks were clearly separated from other correlations. X-NR3 (C_3_/H_3_) appeared at δ_C_/δ_H_ 75.4/3.33, and two X-NR5 (C_5_/H_5_) peaks were located at δ_C_/δ_H_ 68.1/3.22 and 3.86. The reducing-terminal-end (α,β) of d-Xylp (X-Rα4, X-Rβ4) appeared with an internal xylan peak (X-I4) at δ_C_/δ_H_ 76.2/3.67 (C_4_/H_4_). In addition, a strong 2-*O*-Ac-*β*-d-Xylp (C_2_/H_2_) correlation at δ_C_/δ_H_ 73.2/4.58 and a 3-*O*-Ac-*β*-d-Xylp (C_3_/H_3_) correlation at δ_C_/δ_H_ 74.9/4.88 can be detected in the spectrum of H170-30, suggesting that acetyl groups still existed in the XOS fractions. However, the signal intensity of the acetylated XOS was decreased with the aggravation of pretreatment severity. Meanwhile, the DP of XOS, which can be calculated from 2D-HSQC spectra by dividing the integrals from anomeric signals (X1) of xylp residues by those from reducing-end xylp residues, was decreased from 12.42 in H170-30 to 3.28 in H170-60, and further to 1.49 in H180-30.

### 3.3. The Effect of Hydrothermal Pretreatment on the Lignin Structure

The molecular weights and polydispersity (M_w_/M_n_) of all the acetylated DELs were analyzed by GPC to provide the indirect information of lignin depolymerization and recondensation during hydrothermal pretreatment, and the results are shown in Table 3. The M_w_ of DEL prepared from *TL* was 6690 g/mol, while the M_w_ of PDELs obtained from hydrothermal *TL* was significantly reduced to 3500–4350 g/mol. In addition, the M_w_ was further decreased with prolonged pretreatment time and elevation of temperature. This is probably related to the depolymerization of lignin, such as cleavage of β-*O*-4 linkages. Moreover, the relatively higher molecular weights of DEL are related to the increasing of hydrodynamic volume of lignin linked by carbohydrates [33]. Furthermore, all the PDELs showed relatively lower M_w_/M_n_ values ranging from 1.83 to 1.52, implying that a relatively homogeneous lignin fraction could be obtained under harsh hydrothermal condition (180 °C for 30 min).

**Figure 3:**
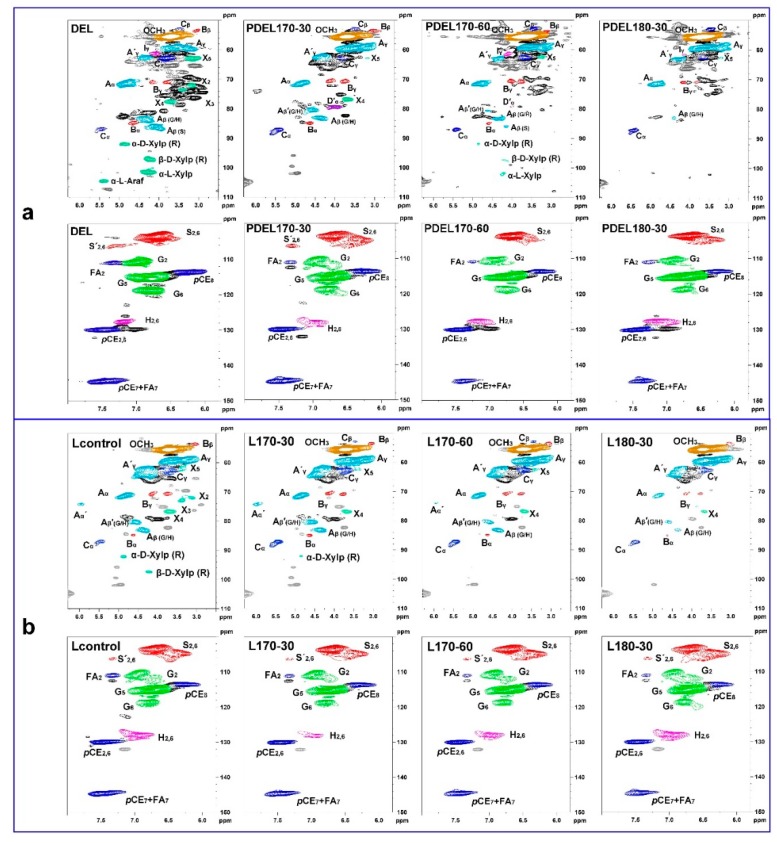
2D-HSQC spectra of enzymatic (**a**) and extracted (**b**) lignin fractions.

2D-HSQC NMR was used to identify the detailed chemical structures and substructures presented in the lignin samples. The correlated signals were assigned as previously described in [34]. In the side-chain regions of the 2D-HSQC spectra (Figure 3a), the spectrum of DEL showed common signals for lignin and carbohydrates. In the spectrum, the residue of xylan backbone X-I2 (C_2_/H_2_), X-I3 (C_3_/H_3_), X-I4 (C_4_/H_4_), and X-I5 (C_5_/H_5_) showed some distinct peaks. In addition, the reducing-end of *β*-d-Xylp was observed at δ_C_/δ_H_ 97.2/4.22, and the correlated *α*-d-Xylp reducing-end was identified at about δ_C_/δ_H_ 92.0/4.87. The *α*-l-Araf was observed at δ_C_/δ_H_ 104.5/5.40. However, these signals for carbohydrates weakened or vanished in the spectra of the PDELs which were prepared from the hydrothermally-treated substrates, suggesting that the carbohydrates (mainly xylans) were heavily removed after the hydrothermal pretreatment. This was in agreement with the content of carbohydrates in lignin, and verified the connection between carbohydrates with the decrease of lignin molecular weight. In addition to the signals for xylan, lignin presented the most prominent signals. The signals of main substructures, such as β-*O*-4 aryl ethers (A), resinols (B), and phenylcoumarans (C), were mostly remarkable in the spectra of DEL. However, some signals of substructures were weakened after different hydrothermal pretreatments. To investigate the effects of hydrothermal pretreatments on the structural changes of lignin, the quantitative results based on 2D-HSQC were calculated and listed in Table 4. It was found that the contents of β-*O*-4 and β-β linkages were decreased in the PDELs as compared with those of DEL, indicating that the cleavage of these linkages could take place under the hydrothermal process [35,36]. The content of β-*O*-4 and β-β linkages was further reduced with the intensification of reaction conditions, which finally led to the decrease of M_w_ of the lignin. The content of β-5 linkage was decreased from 1.03/100Ar in DEL to 0.44/100Ar in PDEL170-30, and increased to 1.03/100Ar in PDEL170-60 and 1.33/100Ar in PDEL180-30, implying that the cleavage of β-5 probably took place at 170 °C for 30 min. Further, repolymerization is more serious than depolymerization at more severe condition.

**Figure 4:**
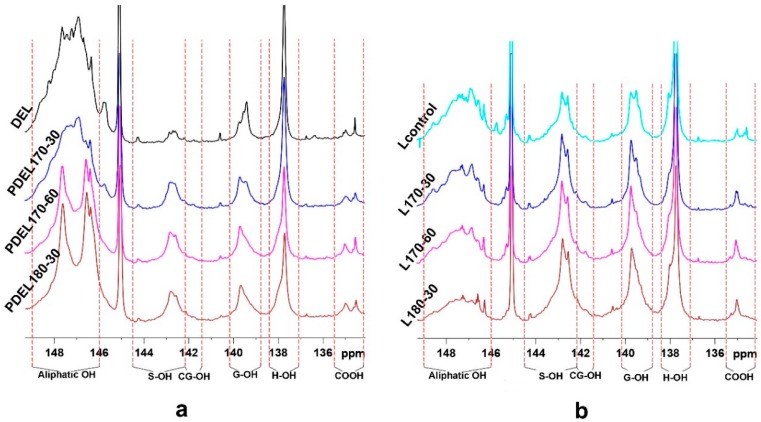
The qualitative analysis of various hydroxyl groups in enzymatic (**a**) and extracted (**b**) lignin by ^31^P-NMR spectra.

In the aromatic region, signals from syringyl (S), guaiacyl (G), *p*-hydroxyphenyl (H), *p*-coumarate (*p*CE), and ferulate (FA) units can be detected. However, the intensity of some signals in the PDELs was weaker than that in DEL, indicating that some linkages were cleaved during the hydrothermal process. In detail, the contents of FA_2_ and *p*CE_2,6_ substructures units were reduced from 4.79/100Ar in DEL to 4.08/100Ar in the PDEL170-30, suggesting that the linkages between lignin and *p*CE/FA were cleaved under the hydrothermal pretreatment. Meanwhile, their contents were further decreased with prolonged pretreatment time and elevation of temperature. Additionally, S/G ratio is important to evaluate the structural features of lignin. The S/G ratio of DEL was 0.94, and that of PDELs were ranged from 1.22 to 1.83. The increased S/G ratio of the substrates after the hydrothermal pretreatment suggests that G-type lignin units were enriched in the raw materials *TL*, however, the G-type lignin units were readily fragmented and released from the biomass during the hydrothermal process [37,38], thus, the S/G ratio of lignin in the hydrothermally-treated substrates was increased. The contents of various hydroxyl groups in these DEL samples are essential to investigate the effects of hydrothermal pretreatment on the structural features of lignin. The quantitative ^31^P NMR technique was applied to the enzymatic lignin samples and the corresponding results are summarized in Figure 4a and Table 5. As shown in Table 5, the content of aliphatic OH was reduced after hydrothermal pretreatment as compared with that (4.71 mmol/g) of DEL, suggesting that the aliphatic OH groups in carbohydrates were removed after the hydrothermal process, which is in agreement with the carbohydrate analysis of the DELs samples (Table 1). However, the content of aliphatic OH increased from 3.27 mmol/g in PDEL170-30 to 3.32 mmol/g in PDEL170-60 to 3.80 mmol/g in PDEL180-30, implying that the cleavage of some lignin-carbohydrates complex (LCC) occurred in the hydrothermal pretreatment, and aliphatic OH in the side-chain in lignin was released with the extension of hydrothermal pretreatment time and the rise of reaction temperature [39]. In addition, the content of S and G-type phenolic OH groups were obviously increased after the hydrothermal pretreatment, which is mostly ascribed to the cleavage of β-*O*-4 linkages as also reflected by the changes of linkages in 2D HSQC spectra (Table 4). Moreover, the contents of H-Type phenolic OH groups (mainly originated from *p*CE) were slightly reduced from PDEL170-30 to PDEL180-30, suggesting that the esterified *p*CE was slightly cleaved by the enhancement of reaction time and temperature during hydrothermal process. Furthermore, the contents of COOH groups in the PDEL samples were increased as compared to that of DEL without hydrothermal pretreatment, suggesting that some aliphatic OH groups oxidized into COOH groups during the pretreatment. In short, the structural changes of lignin induced by the hydrothermal pretreatment will affect the subsequent organic acid delignification process. 

### 3.4. The Effect of Hydrothermal Pretreatment on the Structural Features of Organosolv Lignin

To investigate the effect of hydrothermal pretreatment on the subsequent organic acid (formic acid/acetic acid/water, 3/5/2, *v*/*v*/*v*) delignification, molecular weight, 2D-HSQC, and ^31^P-NMR spectra of organosolv lignin were collected and are depicted in Table 3, Figure 3b and Figure 4b. The substructures in the spectra of lignin were assigned as previous publication [34] and listed in the Supplementary Material, Table S2. 

Comparisons of molecular weights of the control and organosolv lignin can reflect the effects of hydrothermal pretreatment on the structural features of organosolv lignin. The M_w_ of organosolv lignin obtained from hydrothermal *TL* ranged from 2290 g/mol in L180-30 to 3870 g/mol in L170-30, compared with that (4200 g/mol) of Lcontrol, suggesting that different degrees of cleavage of lignin substructures occurred during the hydrothermal pretreatment, which can also be verified by the structural changes of lignin. As shown in Figure 3b, β-aryl ether (A), resinol (B), phenylcoumaran (C), and methoxyl groups (OCH_3_) are readily identified in the side-chain region of the spectra. Herein, the detailed assignments are plotted in the spectra in different colors as in the aforementioned NMR section. Quantitative results demonstrate that the content of β-*O*-4 linkages in organosolv lignin was significantly reduced (15.66–2.69/100Ar) as compared to the corresponding DEL (49.96/100Ar) and PDELs (12.06–33.03/100Ar). This suggests that subsequent organic acid delignification results in the cleavage of β-*O*-4 linkages in lignin. In addition, the content of β-*O*-4 linkage in the organosolv lignin (L170-30, L170-60, L180-30) was decreased from 12.88 to 2.69/100Ar as the enhancement of pretreatment severity increases. Interestingly, the acetylation reaction also occurred at α-position (δ_C_/δ_H_ 74.2/5.95) of β-*O*-4 linkage during the organic acid treatment, which is in accordance with previous results [11,15]. In fact, the acetylation reaction was also certified by the newly-presented signal at δ_C_/δ_H_ 80.5/4.56 (A_β_′(G/H)), which is due to the acetylation of β-*O*-4 linkages [40]. Among these organosolv lignin samples, it was found that L170-30 presented the highest degree of acetylation, as revealed by the strongest signals at δ_C_/δ_H_ 74.2/5.95. Higher degree of acetylation is supposed to result in higher lignin hydrophobicity, which can be used in carbon fibers production [41] and lignin-plastic composite [42]. In addition, the acetylated lignin can be also used as a promising material for the catalytic upgrading of lignin [43,44].

In addition to the abundant β-*O*-4 linkages, the resinol (β–β, substructures B) appeared in the spectra in noticeable amounts, labeled as C_α_–H_α_, C_β_–H_β_, and the double C_γ_–H_γ_ correlations at δ_C_/δ_H_ 84.7/4.62, 53.5/3.07, 70.8/3.76, and 4.18, respectively. The content of β-β linkages was decreased from 0.88/100Ar in L170-30 to 0.23/100Ar in L180-30, suggesting that the cleavage of β–β linkages could take place during the hydrothermal pretreatment and organic acid delignification processes. By contrast, the content of β-5 linkages was elevated in organosolv lignin as compared to that of DEL, suggesting that β-5 linkages could be possibly formed during delignification process [35,36]. In general, the M_w_ of lignin was decreased when the depolymerization reactions occur, while condensation reactions usually result in an increased molecular size with heterogeneous lignin structures [45]. Herein, the M_w_ of organosolv lignin was decreased, suggesting the dominant role of depolymerization over condensation under these hydrothermal conditions.

In the aromatic regions (δ_C_/δ_H_ 100.0–150.0/5.80–8.00) of 2D-HSQC spectra (Figure 3b (bottom)), the signals of the syringyl (S), guaiacyl (G), and *p*-hydroxyphenyl (H) were distinctly observed. After the organic acid treatment, it should be noted that obvious condensation signals of S_2,6_ and G_2_ appeared at δ_C_/δ_H_ 103.6/6.70 and 110.7/6.95, respectively. The other correlations of S and G units signals observed at δ_C_/δ_H_ 106.2/7.34, 115.5/6.76, and 118.8/6.78 were assigned to the Cα-oxidized S-units (S′_2,6_), G_5_, and G_6_ units, respectively. Interestingly, the C_2,6_–H_2,6_ correlations of H unit presented a stronger signal in organosolv lignin than that in the corresponding DELs. The S/G ratio of lignin also increased from 0.94 in DEL to 1.22–1.83 in PDELs, and further to 1.26–1.61 in organosolv lignin samples (L170-30, L170-60, and L180-30). In addition, the signals of FA and *p*CE in the spectra of grasses were observed in all the lignin fractions, as revealed by the signal of *p*CE_8_ (C_8_–H_8_ correlation at δ_C_/δ_H_ 113.5/6.25) [15]. The presence of this signal indicated that the FA and *p*CE were still linked to lignin macromolecule with ester bonds. From the quantitative result in Table 4, the content of *p*CE_2,6_ was decreased from 63.61/100Ar in DEL to 42.60/100Ar in Lcontrol indicating that the linkage between *p*-coumaric acid and lignin was cleaved under the delignification process. Meanwhile, the corresponding content was further decreased to 18.74/100Ar in L180-30 with the treatment of the hydrothermal process, implying that the linkage could be easily cleaved in the hydrothermal and organosolv delignification.

The quantitative results of hydroxyl groups in organosolv lignin samples (Figure 4b and Table 5) could also reflect the structural changes during the proposed pretreatment. In detail, the content of aliphatic OH groups ranged from 1.80 mmol/g in Lcontrol to 1.06 mmol/g in L180-30, which is significantly lower than that (4.71 mmol/g) of DEL. The change of aliphatic OH groups is attributed to the oxidization during the hydrothermal process and organic acid treatment. Additionally, the contents of S, G, and H-type phenolic OH groups were significantly increased with the organic acid treatment, especially for the high temperature and prolonged reaction time. However, no obvious differences among COOH groups and H-type OH groups were observed.

### 3.5. Mass Balance of the Hydrothermal and Organic Acid Delignification Process

The combined process of hydrothermal pretreatment and organic acid delignification was applied to *TL*, and mass balance of the hydrothermal and organic acid delignification process was calculated based on dried weight of *TL* (Figure S1). Although the experiment was performed at laboratory scale, the calculation of mass balance based on dried weight of raw material can intuitively display the desired effect of the combined process. The main products obtained after hydrothermal pretreatment are xylooligosaccharides (XOS, DP 2–6 and DP > 6), which yielded 3.04–13.55%. After the organic acid delignification, 13.77–17.59% acetylated lignin could be obtained. In addition, 30.47–39.04% cellulose-rich residue was obtained. In short, under the optimal pretreatment condition (170-30), it was found that 13.55% XOS, 14.15% acetylated lignin, and 39.04% crude pulp (cellulose-rich residue), could be successively obtained from oven-dried *TL* as pretreated by the integrated process.

## 4. Conclusions

*Triarrhena lutarioriparia* was successfully fractionated into XOS, acetylated lignin, and cellulose-rich substrates based on the hydrothermal and organic acid delignification. Under mild hydrothermal pretreatment, hemicelluloses in the raw material were depolymerized into XOS with different degrees of polymerization in a high yield (13.55% based on dried weight of *TL*). Structural changes of lignin during the hydrothermal pretreatment also facilitated organic acid delignification, in which simultaneous dissolution and derivatization of lignin occurred in a high-efficiency approach. The acetylated lignin obtained can be used as a promising material for lignin valorization, such as catalytic degradation of lignin and lignin-plastic composite. 

## Figures and Tables

**Table 1:** The carbohydrates analysis of the lignin obtained under different conditions.

Name	Sugar/%	Ara ^a^	Gal ^a^	Glu ^a^	Xyl ^a^	Man ^a^	Glua ^a^
DEL	18.42	1.90	0.40	4.05	10.14	1.32	0.61
PDEL170-30	7.87	0.56	0.37	4.52	1.69	0.45	0.28
PDEL170-60	7.41	0.11	0.33	5.33	0.54	0.86	0.24
PDEL180-30	7.99	0.03	0.24	6.02	0.32	1.17	0.21
Lcontrol	2.80	0.84	0.06	0.11	1.32	0.20	0.27
L170-30	2.95	0.74	0.03	0.31	1.26	0.39	0.22
L170-60	2.50	0.67	0.25	0.25	0.65	0.51	0.17
L180-30	2.19	0.43	0.27	0.27	0.23	0.93	0.06

^a^ Ara, arabinose; Gal, galactose; Glu, glucose; Xyl, xylose; Man, Mannose; Glua, glucuronic acid.

**Table 2:** The degraded products of *Triarrhena lutarioriparia* (*TL*) during the different hydrothermal process (g/L).

Conditions	Formic Acid	Acetic Acid	Lactic Acid	Furfural	HMF
170-30	0.30	1.67	0.35	0.12	0.08
170-60	1.17	2.52	1.08	3.24	0.21
180-30	1.34	3.86	1.29	4.09	0.41

**Table 3:** The gel permeation chromatography (GPC) analysis of enzymatic and extracted lignin samples.

	M_w_	M_n_	M_w_/M_n_
DEL	6690	3800	1.76
DEL170-30	4180	2290	1.83
DEL170-60	4350	2720	1.60
DEL180-30	3500	2300	1.52
Lcontrol	4200	2080	2.02
L170-30	3870	1930	2.01
L170-60	3450	1830	1.89
L180-30	2290	1630	1.40

**Table 4:** Quantification of the enzymatic and extracted lignin fractions by 2D heteronuclear single quantum coherence (2D-HSQC) NMR spectra.

	β-*O*-4	β-5	β-β	FA_2_	*p*CE_2,6_	S/G ^b^
DEL	49.96 ^a^	1.03	1.37	4.79	63.61	0.94
PDEL170-30	33.03	0.44	0.22	4.08	59.07	1.33
PDEL170-60	19.10	1.03	0.03	2.50	41.25	1.22
PDEL180-30	12.06	1.33	ND	3.85	48.19	1.83
Lcontrol	15.66	3.31	0.78	3.31	42.60	1.54
L170-30	12.88	3.62	0.88	3.17	31.68	1.30
L170-60	8.92	3.93	0.36	2.57	24.04	1.61
L180-30	2.69	2.49	0.23	1.63	18.74	1.26

^a^ Results expressed per 100 Ar based on quantitative 2D-HSQC spectra. ^b^ S/G ratio obtained by the Equation: S/G ratio = 0.5I (S_2,6_)/I (G_2_).

**Table 5:** Quantification of different OH groups in the enzymatic and extracted lignin fractions by quantitative ^31^P-NMR method (mmol/g).

Sample	Aliphatic OH	Syringyl OH	Guaiacyl OH		*p*-Hydroxyphenyl OH ^c^	Carboxylic Group
			C ^a^	NC ^b^		
DEL	4.71	0.28	0.07	0.48	0.82	0.29
PDEL170-30	3.27	0.52	0.11	0.50	0.83	0.30
PDEL170-60	3.32	0.60	0.12	0.58	0.77	0.38
PDEL180-30	3.80	0.58	0.12	0.58	0.77	0.39
Lcontrol	1.80	0.80	0.14	0.68	1.06	0.20
L170-30	1.68	1.06	0.16	0.79	1.05	0.22
L170-60	1.40	1.22	0.20	0.91	1.15	0.24
L180-30	1.06	1.33	0.22	0.97	1.16	0.26

^a^ C, condensed. ^b^ NC, non-condensed. ^c^ *p*-hydroxyphenyl OH contains *p*-coumarate OH and *p*-hydroxyphenyl OH groups.

## Supplementary Materials

Supplementary data associated with this article can be found, in the online version, at http://www.mdpi.com/2073-4360/10/10/1157/s1. Table S1: The abbreviations and elaboration of products obtained from hydrothermal and organic acid treatment; Table S2: Assignments of ^13^C-^1^H correlated signals in the HSQC spectra of the lignin; Figure S1: Mass balance of the hydrothermal and organic acid delignification process.
