# Supplementary material for: Effects of Hydrothermal Pretreatment on the Structural Characteristics of Organosolv Lignin from Triarrhena lutarioriparia
Source: Polymers (Basel). 2018 Oct 16;10(10):1157. doi: 10.3390/polym10101157 (PMC6403627; doi:10.3390/polym10101157)

Electronic Supplementary Information

Effects of hydrothermal pretreatment on the structural characteristics of organosolv lignin from Triarrhena lutarioriparia

Tianying Chen[a], Zhiwen Li[a], Xueming Zhang[a], Douyong Min[b], Yuying Wu[a], Jialong Wen[a],* Tongqi Yuan[a]

[a] Beijing Key Laboratory of Lignocellulosic Chemistry, Beijing Forestry University, Beijing 100083, China

[b] Guangxi Key Laboratory of Clean Pulp & Papermaking and Pollution Control, Nanning, 530004, China

*Corresponding authors. Address:

Beijing Key Laboratory of Lignocellulosic Chemistry, Beijing 100083, China.

Tel: +86-10-62336903; fax: +86-10-62336903 (J.L. Wen).

E-mail address: wenjialonghello@126.com (J.L. Wen)

The acetylation of lignin

50 mg of lignin sample was dissolved in a 3 mL solution of DMSO: N-Methylimidazole (2: 1, v/v). The dissolution was performed on a shaker at room temperature for 24 h in the dark. After that, 1 mL of acetic anhydride was added into the solution for an additional 1.5 h. At the end of the designated time, the solution was dropped slowly into 300 mL of acid water (pH = 2.0) adjusted by HCl to induce precipitation, and the precipitate was washed thoroughly with acid water (pH = 2.0) until significant reducing of pungent odor, and then freeze-dried to obtain the acetylated lignin. Extracted lignin samples were synchronous acetylated, which can be well compared with acetylated DELs.

The calculation of severity log R0

In this section, the severity of hydrothermal treatment was mentioned in terms of the severity log R_0, which quoted the results of Wang 2016 to demonstrate that the oligosaccharides were mainly released among the hydrothermal conditions in this study.

Wang et al reported that the severity log R_0 was calculated by the following formula according to previous paper [1],

$$\log R_0 = \log\ [R_{0\mathrm{HEATING}} + R_{0\mathrm{ISOTHERMAL}} + R_{0\mathrm{COOLING}}]$$

$$= [\log \int_0^{t_H} \exp \left(\frac{T\,(t)\text{-}T_{\mathrm{REF}}}{\omega}\right) \cdot \mathrm{d}t + t \cdot \exp\left(\frac{T\,(t)\text{-}T_{\mathrm{REF}}}{\omega}\right) + \int_0^{t_C} \exp\left(\frac{T\,(t)\text{-}T_{\mathrm{REF}}}{\omega}\right) \cdot \mathrm{d}t]$$

where t_H (min) is the time needed to achieve the target temperature, t_C (min) is the time needed for the whole heating–cooling period, t (min) is the retention time, and $T(t)$ represents the treatment temperature (°C). Calculations were made based on the values reported in the literature (ω and T_{REF} are 14.75 and 100 °C, respectively) [1, 2].

References

[1] Overend, R.P.; Chornet, E.; Gascoigne, J.A. Fractionation of Lignocellulosics by Steam-Aqueous

Pretreatments [and Discussion]. *Philos Trans R Soc Lon A Math Phys Eng Sci*. **1987**, *321*, 523-36; DOI: 10.1098/rsta.1987.0029.

[2] Kim, Y.M.; Kreke, T.; Mosier, N.S.; Ladisch, M.R. Severity factor coefficients for subcritical liquid hot water pretreatment of hardwood chips. Biotechnol Bioeng. **2013**, *111*, 254-63; DOI: 10.1002/bit.25009.

Table S1. The abbreviations and elaboration of product obtained from hydrothermal and organic acid treatment

Name	Add hydrothermal process	Reaction temperature (°C)	Reaction time (min)	Description
H170-30	Yes	170	30	The liquid product obtained from hydrothermal process
H170-60	Yes	170	60	The liquid product obtained from hydrothermal process
H180-30	Yes	180	30	The liquid product obtained from hydrothermal process
P170-30	Yes	170	30	The residue obtained from hydrothermal pretreatment
P170-60	Yes	170	60	The residue obtained from hydrothermal pretreatment
P180-30	Yes	180	30	The residue obtained from hydrothermal pretreatment
Ocontrol	No	-	-	The residue obtained from direct organic acid treatment of TL
OP170-30	Yes	107	180	The residue obtained from organic acid treatment of P170-30
OP170-60	Yes	107	180	The residue obtained from organic acid treatment of P170-60
OP180-30	Yes	107	180	The residue obtained from organic acid treatment of P180-30
Lcontrol	No	107	180	The lignin obtained from organic acid treatment of TL
L170-30	Yes	107	180	The lignin obtained from organic acid treatment of P170-30
L170-60	Yes	107	180	The lignin obtained from organic acid treatment of P170-60
L180-30	Yes	107	180	The lignin obtained from organic acid treatment of P180-30

All the samples were soaked in organic acid (formic acid/ acetic acid/water, 3/5/2, v/v/v)

at 60 °C for 1 h before treated with organic acid.

Table S2. Assignments of ^{13}C-^{1}H correlated signals in the HSQC spectra of the lignin

Labels	δ_C/δ_H (ppm)	Assignment
C_β	52.9/3.47	C_β-H_β in phenylcomaran structures(C)
B_β	53.5/3.07	C_β-H_β in resinol substructures(B)
$-OCH_3$	55.4/3.71	C-H in methoxyls
A_γ	59.8/3.59	C_γ-H_γ in β-*O*-4 substructures(A)
I_γ	61.3/4.08	C_γ-H_γ in cinnamyl(sinapyl/coniferyl) alcohol end groups(I)
X_5	62.8/3.12	C_5-H_5 β-D-xylopyranoside substructures(X)
A'_γ	62.9/4.27	C_γ-H_γ in γ-acylated β-*O*-4 substructures(A')
B_γ	70.8/3.76 and 4.18	C_γ-H_γ in resinol substructures(B)
A_α	71.8/4.85	C_α-H_α in β-*O*-4 substructures(A)
A'_α	74.2/5.95	C_α-H_α in α-acylated β-*O*-4 substructures(A')
A'_β(G/H)	80.5/4.56	C_β-H_β in β-*O*-4 linked to a G/H unit(A)
A_β(G/H)	83.3/4.35	C_β-H_β in β-*O*-4 linked to a G/H unit(A)
B_α	84.7/4.62	C_α-H_α in resinol substructures(B)
A_β(S)	85.9/4.11	C_β-H_β in β-*O*-4 linked to a S unit(A)
C_α	86.9/5.47	C_α-H_α in phenylcomaran structures(C)
$S_{2,6}$	103.6/6.70	$C_{2,6}$-$H_{2,6}$ in syringyl units(S)
$S'_{2,6}$	106.2/7.34	$C_{2,6}$-$H_{2,6}$ in oxidized(C=O) phenolic syringyl units(S')
G_2	110.8/6.96	C_2-H_2 in guaiacyl units(G)
FA_2	110.9/7.28	C_2-H_2 in ferulate (FA)
pCE_8	113.5/6.25	C_8-H_8 in *p*-coumarate (*p*CE)
G_5	115.5/6.76	C_5-H_5 in guaiacyl units(G)
G_6	118.8/6.78	C_6-H_6 in guaiacyl units(G)
$H_{2,6}$	127.7/7.18	$C_{2,6}$-$H_{2,6}$ in *p*-hydroxyphenyl units(H)
$pCE_{2,6}/pCA_{2,6}$	129.9/7.45	$C_{2,6}$-$H_{2,6}$ in *p*-coumarate (*p*CE)
pCE_7/pCA_7	144.4/7.39	C_7-H_7 in *p*-coumarate (*p*CE)

Fig. S1. Mass balance of the hydrothermal and organic acid delignification process

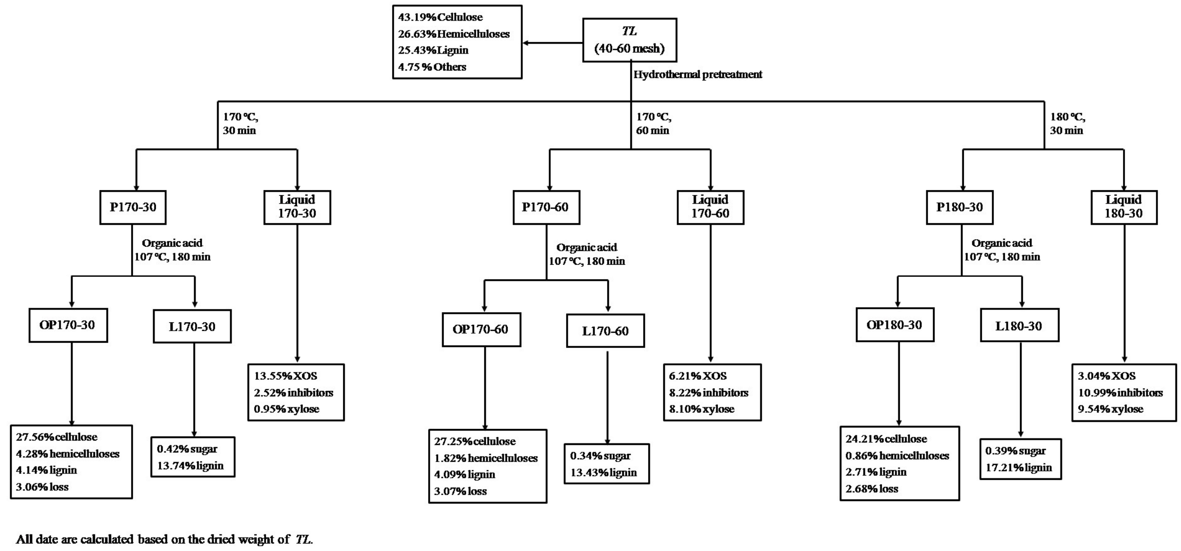

Supplement: Supplementary file 1 [file polymers-10-01157-s001.pdf]
